# The Predictive Value of the Perivascular Adipose Tissue CT Fat Attenuation Index for Coronary In-stent Restenosis

**DOI:** 10.3389/fcvm.2022.822308

**Published:** 2022-04-26

**Authors:** Bin Qin, Zhengjun Li, Hao Zhou, Yongkang Liu, Huiming Wu, Zhongqiu Wang

**Affiliations:** Department of Radiology, Affiliated Hospital of Nanjing University of Chinese Medicine, Nanjing, China

**Keywords:** computed tomography angiography (CTA), perivascular adipose tissue (PVAT), fat attenuation index (FAI), in-stent restenosis (ISR), stents

## Abstract

**Objectives:**

To investigate the association between the perivascular adipose tissue (PVAT) fat attenuation index (FAI) derived from coronary computed tomography angiography (CCTA) and the prevalence of in-stent restenosis (ISR) in patients with coronary stent implantation.

**Methods:**

A total of 117 patients with previous coronary stenting referred for invasive coronary angiography (ICA) were enrolled in this retrospective observational analysis. All patients underwent CCTA between July 2016 and November 2021. The deep learning-based (DL-based) method was used to analyze and measure the peri-stent FAI value. Additionally, the relationship between hematological and biochemical parameters collected from all the patients was also explored. The least absolute shrinkage and selection operator (LASSO) method was applied to the most useful feature selection, and binary logistic regression was used to test the association between the selected features and ISR. The predictive performance for ISR of the identified subgroups was evaluated by calculating the area under the curve (AUC) of receiver operator curves plotted for each model. The Pearson correlation coefficient was used to assess the correlation of peri-stent FAI values with degrees of ISR.

**Results:**

The peri-stent FAI values in the ISR group were significantly higher than those in the non-ISR group (−78.1 ± 6.2 HU vs. −87.2 ± 7.3 HU, *p* < 0.001). The predictive ISR features based on the LASSO analysis were peri-stent FAI, high-density lipoprotein cholesterol (HDL-C), apolipoprotein A1 (ApoA1), and high-sensitivity c-reactive protein (hs-CRP), with an AUC of 0.849, 0.632, 0.620, and 0.569, respectively. Binary logistic regression analysis determined that peri-stent FAI was uniquely and independently associated with ISR after adjusting for other risk factors (odds ratio [OR] 1.403; 95% CI: 1.211 to 1.625; *p* < 0.001). In the subgroup analysis, the AUCs of the left anterior descending coronary artery (LAD), left circumflex coronary artery (LCx), and right coronary artery (RCA) stents groups were 0.80, 0.87, and 0.96, respectively. The Pearson's correlation coefficient indicated a term moderately correlation between ISR severity and peri-stent FAI values (*r* = 0.579, *P* < 0.001).

**Conclusions:**

The peri-stent FAI can be used as an independently non-invasive biomarker to predict ISR risk and severity after stent implantation.

## Introduction

Percutaneous coronary intervention (PCI) with stent placement is a standard revascularization procedure for coronary artery disease (CAD) with significant flow stenosis (1). However, in-stent restenosis (ISR) reduces the overall efficacy of PCI. Despite the development of drug-eluting stents (DES), ISR can still lead to recurrent angina requiring additional target lesion revascularization (TLR) surgery (2, 3). A study by James et al. demonstrated that about 20% to 35% of patients with bare-metal stents (BMS) and 5% to 10% of patients with DES are diagnosed with ISR (4).

Although the cause of ISR is still challenging to elucidate, inflammation is believed to play an important role in its development and progression (5, 6). After stent placement into the coronary arteries, endothelial damage occurs in the blood vessel resulting in a substantial inflammatory response to wound healing. This stimulates vascular smooth muscle cell proliferation and extracellular matrix deposition, followed by neointimal thickening, granulation formation, and restenosis (6–8). Furthermore, perivascular adipose tissue (PVAT) also plays a pivotal role in regulating vascular homeostasis and disease. PVAT is defined as respective perivascular adipose tissue within a radial distance from the outer vessel wall equal to the diameter of the coronary artery (9). PVAT participates in complicated bidirectional interactions with adjacent vessel walls (9–13) whereby the inflamed coronary arteries release and diffuse the related signals to the PVAT, thereby inhibiting local adipogenesis (9).

The fat attenuation index (FAI) was developed as an imaging biomarker to capture and quantify changes in the attenuation of perivascular fat by inflammation, enabling early detection of coronary inflammation using conventional methods (9). Standard coronary computed tomography angiography (CCTA) allows for a three-dimensional radiomic texture assessment of the PVAT spatial gradients induced by inflammation by mapping the weighted attenuation gradients (9). It has been demonstrated that perivascular FAI based on traditional CCTA measurements can be used as a non-invasive biomarker of coronary artery inflammation, with a higher prognostic value than anatomical coronary artery stenosis or calcification (14). Moreover, further studies have confirmed that the FAI value is associated with the presence of unstable plaques in acute coronary syndromes (15) and can predict the future progression of coronary atherosclerotic plaques (16).

Several artificial intelligence (AI)-based approaches have recently been applied to PVAT quantification (17). The integration of machine learning (ML), deep learning (DL), and radiomic methods has meaningful clinical implications by revealing direct links between tissue imaging phenotyping and tissue biology (18). However, to our knowledge, there has been no investigation of the peri-stent FAI in patients who have undergone intracoronary stenting placement previously.

Therefore, this study aimed to investigate the correlation between peri-stent FAI derived from CCTA using a DL-based method with ISR after PCI. Furthermore, we also examine the feasibility of using peri-stent FAI as an independent predictor of ISR.

## Materials and Methods

### Study Design and Population

**Figure 1:**
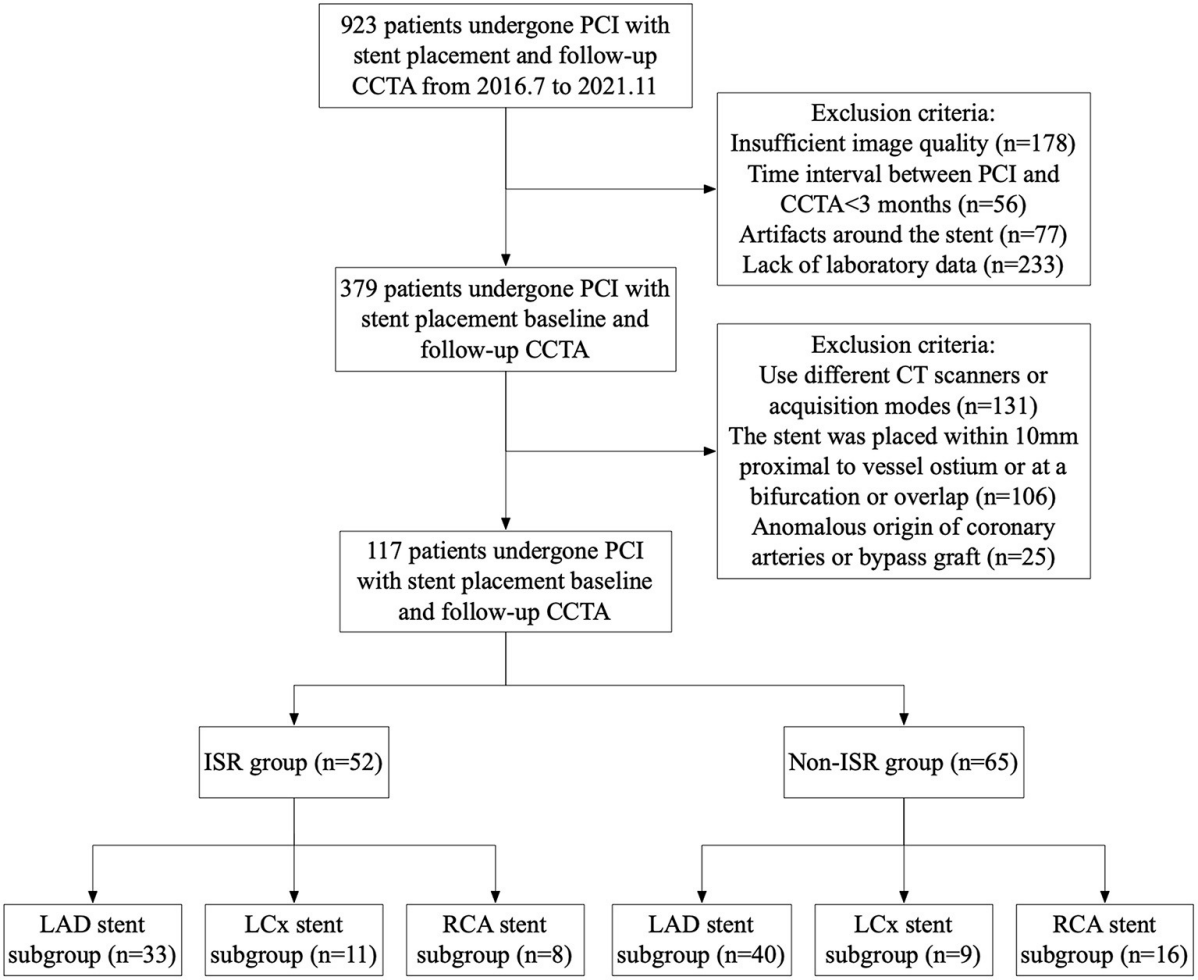
Study flowchart.

The local Medical Ethics Committee review board approved the study, and the requirement for written informed consent was waived for all patients due to the retrospective nature of the study. This study was conducted in line with the declaration of Helsinki. All patients who have undergone follow-up CCTA examination after a period of time of PCI with stenting placement in our hospital between July 2016 and November 2021 were enrolled in the study (Figure 1). The clinical exclusion criteria for the study were a time interval between the PCI and CCTA examination below 3 months; lack of laboratory data, patients with renal failure, a stent positioned within 10 mm proximal to the vessel or ostium, stents located at a bifurcation, overlapping stents, an anomalous coronary artery origin, and the presence of a coronary artery bypass graft. Patients whose CT images were acquired using CT scanners from different manufacturers and acquisition modes were excluded. Patients whose images could not be used to determine the presence of ISR formation within the stent lumen due to poor image quality or the presence of artifacts were not included in the study. Additionally, patients who had artifacts around the stent that affected the accuracy of the FAI measurement were also excluded. Only one stent per patient was included in the analysis. If a patient had multiple coronary stent stenosis, the most severe one was included in the ISR group. Conversely, if a patient had numerous stents without stenosis, the most visible one was included in the control group. Relevant demographics and medical history were obtained at baseline.

### Laboratory Analysis

Routine blood tests were obtained from all participants within three days before and after the CCTA examination at our hospital's center. All blood samples were obtained in the morning after an overnight fast of more than 12 h. The hematological and biochemical parameters analyzed included; complete blood cell count; hemoglobin; total bilirubin, direct bilirubin, and indirect bilirubin; creatinine and creatine phosphokinase (CPK), brain natriuretic peptide (BNP), total cholesterol, high-density and low-density lipoprotein cholesterol (HDL-C and LDL-C), triglycerides (TG), apolipoprotein A1, B and E (ApoA1, ApoB, and ApoE); red cell distribution width (RDW), high-sensitivity c-reactive protein (hs-CRP), and hemoglobin A1c (HbA1c).

### CCTA Protocol and Image Reconstruction

All CCTA images were acquired on a 256-slice computed tomography scanner (Brilliance iCT, Philips Healthcare, Amsterdam, The Netherlands). β-blocker (25–75 mg) was administered orally 1 h before the examination for patients with a resting heart rate (HR) above 65 beats per minute (bpm) to achieve the target HR of 65 bpm or less. Sublingual nitroglycerin (0.5 mg) was given to all patients for coronary vasodilation while the scan was being performed. A prospective electrocardiogram (ECG) triggering acquisition protocol was adopted. The parameters used were a tube voltage of 120 kV, an effective tube current-time product of 600 mAs with ECG-dependent tube current modulation, a pitch of 0.18, a gantry rotation time of 270 ms, and a detector collimation of 128 × 0.625 mm. A 370 mg iodine/mL bolus of non-ionic iodinated contrast media (iopromide) (Ultravist® Bayer Healthcare, Berlin, Germany) was injected intravenously at a rate of 4.5–6.5 mL/s [according to the body mass index (BMI)], followed by 20–40 mL saline flush. The bolus-tracking technique was used to trigger the scan process. All CCTA data were transferred to the Extended Brilliance Workspace (EBW) and the Philips Healthcare computer workstation was used for data analysis. Multi-planar, curved-planar, and maximum intensity projection (MIP) images were reconstructed from the CCTA data and used for observing the coronary arteries.

### Image Analysis

The detected target vessels included the left anterior descending coronary artery (LAD), the left circumflex coronary artery (LCx), and the right coronary artery (RCA). Each vessel was evaluated by two experienced cardiac radiologists (BQ and HZ, with 12 years and 8 years of experience in interpreting cardiac imaging, respectively) who were blinded to the clinical results of the patients. The definition of ISR is recurrent diameter stenosis at the stent segment or its edges (5 mm segments adjacent to the stent) compared to the normal proximal lumen on images orthogonal to the vessel (19). The stent characteristics, including locations, segments, length, and diameter, were recorded by the radiologists and any disagreement was resolved by consensus. The degrees of ISR were obtained by measure the diameter in a cross-sectional view, and was calculated as (normal proximal lumen diameter-minimal stent segment diameter)/ (normal proximal lumen diameter) ×100%. The mean of the stent length, diameter, and degrees of ISR was calculated according to inter-rater agreement analysis.

**Figure 2:**
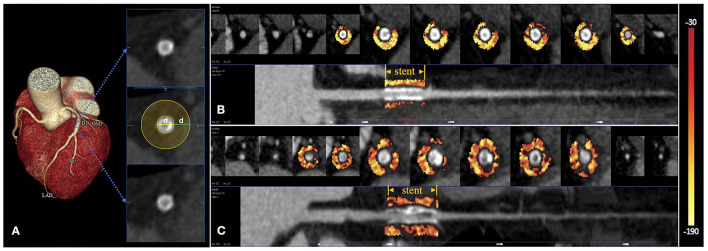
Peri-stent PVAT analysis by DL-based methods. The extent of peri-stent PVAT is visualized in the cross-sectional and straightened views through the adipose tissue Hounsfield unit color table (range −190 HU to −30HU), representing the peri-stent FAI values. **(A)** The peri-stent PVAT was defined as epicardial adipose tissue within a radial distance equal to the diameter of the stent. **(B)** A patient with mid-LAD stent placement. CCTA suggested a non-ISR with a peri-stent FAI of −96 HU. **(C)** Another patient with proximal-LAD stent implantation. ISR can be seen on the CCTA with a peri-stent FAI of −75 HU.

DL-based FAI measurements were carried out on the CCTA analysis platform of Shukun Technology (Perivascular Fat Analysis Tool, Shukun Technology Co., Beijing, China.), using a pre-trained convolutional neural network (CNN) fully automated DL algorithm as follows (Figure 2). First, the CCTA datasets were converted into three-dimensional (3D) volume data, and the coronary arteries were then detected and inputted to a neural network based on a 3D-resnet architecture for structural positioning. The major arteries and branches were then reconstructed and visualized as a multi-planar reformatted volume. Subsequently, we marked the neural network and named each artery, extracted the centerline, and segmented the lumens. Afterward, the cross-sectional image perpendicular to the centerline of the blood vessel was reconstructed, and the attenuation values of the corresponding voxels around the arteries were estimated for the FAI calculation. Finally, the measured area was positioned cover the stent, and the radial distance was manually adjusted so that the diameter was equal to that of the stent. Peri-stent FAI was automatically calculated based on the PVAT attenuation histogram for Hounsfield units (HU) within the range of −190 to −30 HU as previously studied (9). In order to minimize the effects of the aortic wall on FAI outcomes, as previously mentioned, we excluded cases of stents placed within 10 mm of the most proximal coronary arteries (9, 14). The 3D rendered color gradient was used to reflect the FAI value. The two observers performed the FAI measurements independently for all the participants and the mean of FAI was calculated according to inter-rater agreement analysis.

### Statistical Analyses

All statistical analyses were performed on the statistical package for the social sciences (SPSS) statistical software (IBM Co., Armonk, NY, USA) version 26.0, and the R software version 3.6.3 (R Development Core Team). The Prism 9.1.1 software package (GraphPad Prism, San Diego, CA, USA) was used for data presentation. Inter-rater agreement was estimated as Intra-class correlation coefficients (ICC). The least absolute shrinkage and selection operator (LASSO) regression with 10-fold cross-validation was conducted to screen candidate variables with minimum lambda (model 1) and optimal lambda (model 2) to identify the predictors for ISR. The best predictive model was identified by calculating the area under the curve (AUC) of the receiver operative characteristic (ROC) curves plotted for each model. Binary logistic regression analysis determined the association of predictive characteristics with ISR. The Pearson correlation coefficient was calculated to evaluate the relationship between ISR severity and peri-stent FAI values. ROC analysis was also performed to assess the predictive value of selected variables in the diagnosis of ISR and the diagnostic efficacy of peri-stent FAI value for the three major coronary subgroups. A *p*-value of < 0.05 was deemed statistically significant for all statistical tests.

## Results

### Clinical Characteristics of the Patients

A total of 117 patients with coronary artery stent implantations were enrolled in our study, 52 patients were included in the ISR group (mean age: 68.0 ± 11.0 years, 39 males), and 65 patients were included in the non-ISR group (mean age: 66.2 ± 9.9 years, 44 males). There was no significant difference in risk factors and clinical characteristics between the two groups. The baseline and clinical characteristics of the study population are presented in Table 1.

**Table 1:** Baseline characteristics of the patients included.

**Characteristic parameters**	**Non-ISR group (*n* = 65)**	**ISR group** **(*n* = 52)**	***P*** **-value**
Age, years	66.2 ± 9.9	68.0 ± 11.0	0.376
Male gender (%)	44(67.7%)	39(75.0%)	0.387
Risk factors			
Hyperlipidemia (%)	17(26.2%)	15(28.8%)	0.748
Diabetes mellitus (%)	22(33.8%)	18(34.6%)	0.931
Hypertension (%)	31(47.7%)	28(53.8%)	0.512
Smoking (%)	23(35.4%)	21(40.4%)	0.583
Previous myocardial infraction (%)	8(12.3%)	12(23.1%)	0.126
Clinical presentation			
Typical angina (%)	9(13.8%)	7(13.5%)	0.953
Atypical angina (%)	13(20.0%)	10(19.2%)	0.918
Nonspecific chest pain (%)	12(18.5%)	12(23.1%)	0.543
Other (dyspnea, chest discomfort, etc.) (%)	10(15.4%)	9(17.3%)	0.782
Asymptotic (%)	21(32.3%)	14(26.9%)	0.531

*Values are given as mean ± SD (range) or n (%), ISR, in-stent restenosis*.

### Comparison of Laboratory Parameters Between the ISR and Control Groups

The differences in the complete blood cell count, hemoglobin; total bilirubin, direct bilirubin, and indirect bilirubin; creatinine; BNP, total cholesterol, LDL-C, TG, ApoB, ApoE, RDW, hs-CRP, and HbA1c between the two groups were not significant. However, when compared with the control group, the CPK in the ISR group was significantly higher (*p* = 0.034), while the HDL-C (*p* = 0.017) and ApoA1 (*p* = 0.026) in the ISR group were significantly lower. Statistical analyses of hematological and biochemical parameters are described in Table 2.

**Table 2:** Biochemical characteristics of ISR and non-ISR patients evaluated during the angiography.

**Biochemical characteristics**	**Non-ISR group (***n*** = 65)**	**ISR group** **(***n*** = 52)**	***P*** **-value**
Hemoglobin (g/L)	135.3 ± 14.1	131.0 ± 22.2	0.242
Total bilirubin (μmol/L)	10.8 ± 4.5	11.2 ± 4.9	0.691
Direct bilirubin (μmol/L)	3.0 ± 1.4	3.4 ± 1.8	0.144
Indirect bilirubin (μmol/L)	7.8 ± 3.7	7.7 ± 3.4	0.879
Creatinine (μmol/L)	75.4 ± 14.2	83.6 ± 32.0	0.073
CPK (U/L)	93.6 ± 38.3	117.0 ± 72.3	0.034^*^
BNP (pg/ml))	65.2 ± 46.1	199.5 ± 495.1	0.115
Total Cholesterol (mmol/L)	3.5 ± 0.9	3.5 ± 0.9	0.708
HDL-C (mmol/L)	1.3 ± 0.3	1.2 ± 0.2	0.017*
LDL-C (mmol/L)	2.0 ± 0.7	2.0 ± 0.8	0.733
TG (mmol/L)	1.6 ± 1.0	1.4 ± 0.8	0.457
ApoA1 (g/L)	1.2 ± 0.2	1.1 ± 0.2	0.026*
ApoB (g/L)	0.7 ± 0.2	0.7 ± 0.3	0.471
ApoE (mg/dL)	3.6 ± 1.4	3.3 ± 1.1	0.261
Platelet (×10^9^/L)	183.7 ± 43.5	188.5 ± 63.3	0.653
Erythrocytes (×10^9^/L)	4.4 ± 0.4	4.3 ± 0.6	0.381
Leucocytes (×10^9^/L)	6.0 ± 1.6	6.4 ± 2.0	0.255
RDW (%)	12.9 ± 0.9	13.4 ± 1.8	0.063
Lymphocytes (×10^9^/L)	1.7 ± 0.7	1.7 ± 0.7	0.892
Neutrophils (×10^9^/L)	3.5 ± 1.2	3.9 ± 1.6	0.159
Monocytes (×10^9^/L)	0.5 ± 0.2	0.5 ± 0.2	0.678
Hs-CRP (mg/L)	2.8 ± 3.6	4.6 ± 6.6	0.107
HbA1c (%)	6.4 ± 1.2	6.6 ± 1.1	0.385

Values are given as mean ± SD (range), “*” indicate significance at p < 0.05.

*ISR, in-stent restenosis; CPK, creatine phosphokinase; BNP, brain natriuretic peptide; HDL-C, high-density lipoprotein cholesterol; LDL-C, low-density lipoprotein cholesterol; TG, triglycerides; ApoA1, apolipoprotein A1; ApoB, apolipoprotein B; ApoE, apolipoprotein E; RDW, red blood cell distribution width; hs-CRP, high-sensitivity c-reactive protein; HbA1c, hemoglobin A1c*.

### Stent Characteristics and Peri-Stent FAI Values

The ICCs were 0.978 for stent length (95% CI 0.943–0.992, p < 0.001), 0.986 for stent diameter (95% CI 0.965–0.992, *p* < 0.001), 0.927 for degrees of ISR (95% CI 0.838–0.975, *p* < 0.001), correspondingly. There were more cases with LAD stents in both groups, with 33 in the ISR group (63.5%) and 40 in the non-ISR group (61.5%). However, there was no significant difference in the stent locations between the two groups. Among the included cases, the stents located in the distal segment of the coronary artery were less, and most of them were located in the proximal and middle segments. The position, length and diameter of the stents did not differ significantly between the two groups. The DL peri-stent FAI values in the ISR (−78.1 ± 6.2 HU) group were significantly higher when compared with those in the non-ISR group (−87.2 ± 7.3 HU), (*p* < 0.001). The ICC for the peri-stent FAI was 0.985 (95% CI 0.975–0.991, *p* < 0.001), The statistical analyses of the stent characteristics and peri-stent FAI values are summarized in Table 3.

**Table 3:** Stent characteristics and peri-stent PVAT performance between the two groups.

**Parameters**	**Non-ISR group** **(***n***= 65)**	**ISR group** **(***n*** = 52)**	***P*** **-value**
Location of stents			0.347
LAD (%)	40(61.5%)	33(63.5%)	-
LCx (%)	9(13.8%)	11(21.2%)	-
RCA (%)	16(24.6%)	8(15.4%)	-
segments of stents			0.610
Proximal (%)	26(40.0%)	25(48.1%)	-
Middle (%)	36(55.4%)	24(46.2%)	-
Distal (%)	3(4.6%)	3(5.8%)	-
Drug-eluting stent (%)	58(89.2%)	43(82.7%)	0.311
Time period from CCTA to PCI (month)	37.09 ± 14.70	33.85 ± 14.12	0.230
Stent length (mm)	28.0 ± 12.1	28.0 ± 9.8	1.000
Stent diameter (mm)	4.2 ± 0.5	4.2 ± 0.5	0.840
Degree of ISR (%)	50.3 ± 21.5	-	-
Peri-stent FAI (HU)	−87.2 ± 7.3	−78.1 ± 6.2	<0.001***

Values are given as mean±SD (range) or n (%), “***” indicate significance at p < 0.001.

*LAD, left anterior descending coronary artery; LCx, left circumflex coronary artery; RCA, right coronary artery; ISR, in-stent restenosis; FAI, fat attenuation index*.

### Lasso Regression and Binary Logistic Regression Analyses

**Figure 3:**
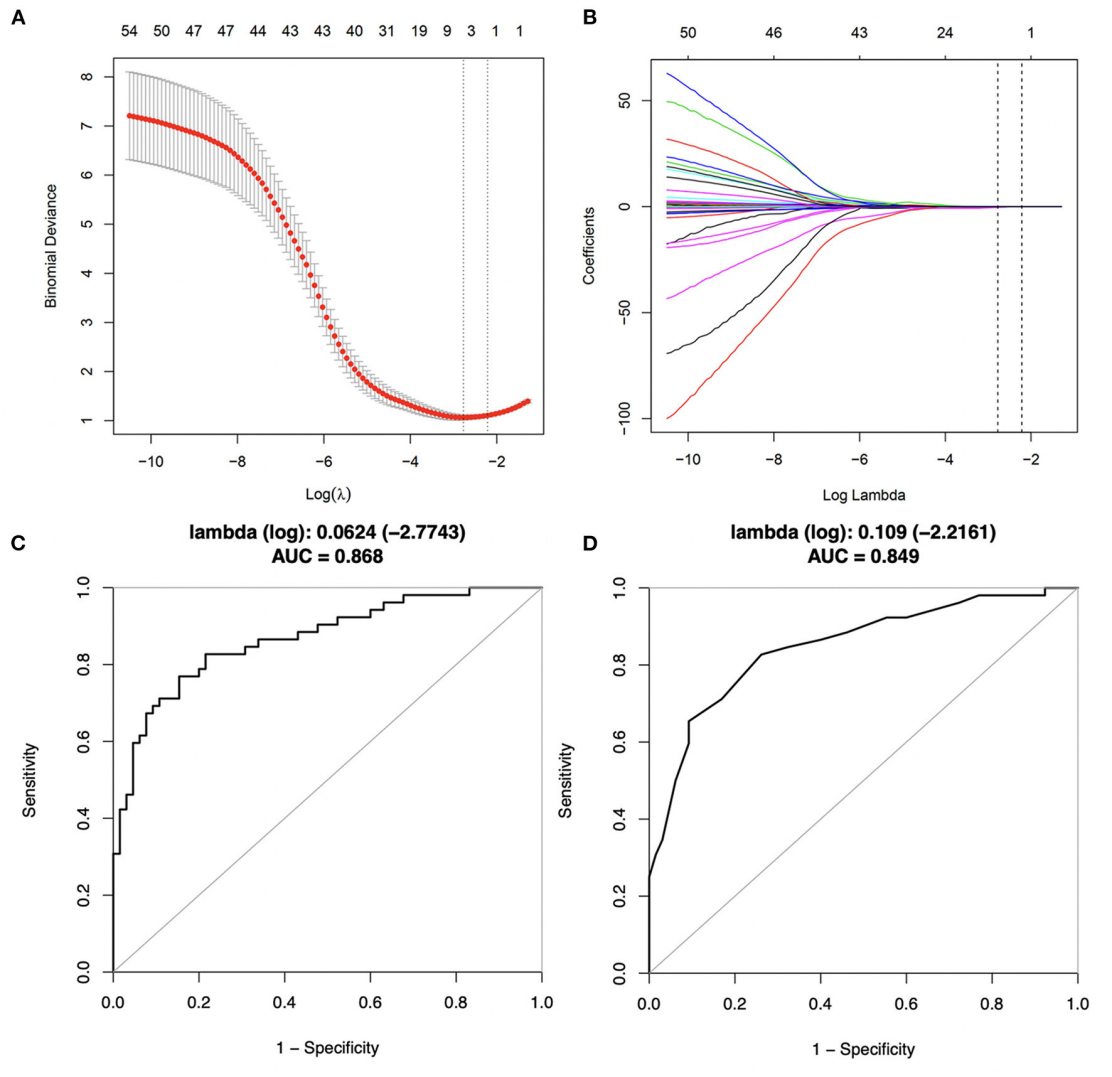
Predictive variables selection using Lasso regression with 10-fold cross-validation. **(A)** Binomial deviance was plotted vs. log (lambda). A total of 46 candidate variables were included. Dotted lines are depicted at the optimal values by minimum criteria (lambda. min, left vertical dotted line) and 1-SE criteria (lambda.1se, right vertical dotted line). **(B)** Coefficients plots were produced against the log (lambda) sequence, profiling all the clinical features. **(C)** Corresponding ROC curve of model 1, with a minimum lambda of 0.0624 and an AUC value of 0.868. **(D)** Corresponding ROC curve of model 2, with an optimal lambda of 0.109 and an AUC value of 0.849.

**Figure 4:**
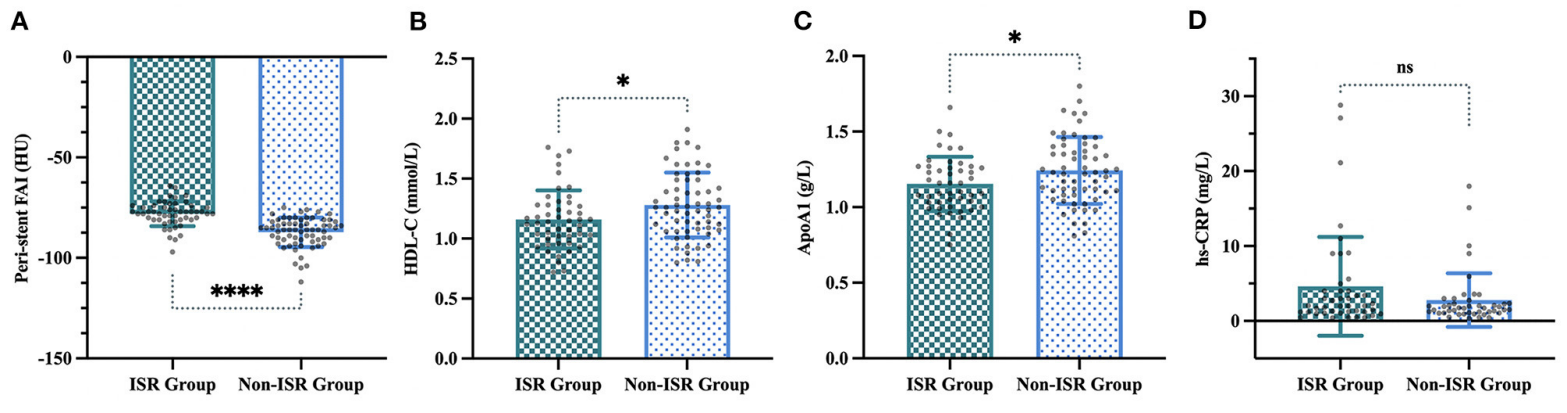
Four variables selected by LASSO regression in the ISR and non-ISR groups. **(A)** Peri-stent FAI values were −78.1 ± 6.2 HU in the ISR group compared with −87.2 ± 7.3 HU in the non-ISR group (*p* < 0.001). **(B)** HDL-C, (1.2 ± 0.2 mmol/L vs. 1.3 ± 0.3 mmol/L, *p* < 0.05). **(C)** ApoA1, (1.1 ± 0.2 g/L vs. 1.2 ± 0.2 g/L, *p* < 0.05). **(D)** Hs-CRP, (4.6 ± 6.6 mg/L vs. 2.8 ± 3.6 mg/L, *p* = 0.107). “*”, “****” and “ns” indicate *p* < 0.05, *p* < 0.001 and non-significant, respectively.

The Lasso regression analysis included all risk parameters mentioned previously (Tables 1–3) and was performed to reduce the risk of overfitting the models and to identify the predictive factors for further analysis (Figure 3). Model 1 included four candidate variables (peri-stent FAI, HDL-C, ApoA1, and hs-CRP) with a minimum lambda of 0.0624, and an AUC of 0.868. On the other hand, model 2 relied on only one candidate variable, i.e., peri-stent FAI, with an optimal lambda of 0.109, and an AUC of 0.849. Based on these findings, we selected peri-stent FAI, HDL-C, ApoA1, and hs-CRP (Model 1) as predictive factors for ISR. In the binary logistic regression analysis, peri-stent FAI was uniquely and independently associated with ISR after adjusting for age, gender, and risk factors (odds ratio [OR] 1.403; 95% CI: 1.211 to 1.625; *p* < 0.001) (Figure 4, Table 4).

**Table 4:** Binary logistic regression analysis for predictors of stent ISR.

**Predictors**	**OR**	**95% CI**	***P*** **-value**
Peri-stent FAI	1.403	1.211–1.625	<0.001***
Hs-CRP	1.066	0.929–1.223	0.364
HDL-C	1.094	0.743–1.611	0.648
ApoA1	0.007	0.000–1.732	0.078

FAI, fat attenuation index; Hs-CRP, high-sensitivity c-reactive protein; HDL-C, high-density lipoprotein cholesterol; ApoA1, apolipoprotein A1. “***” indicate significance at p < 0.001.

### Predictive Performance of the ISR Testing Parameters

**Figure 5:**
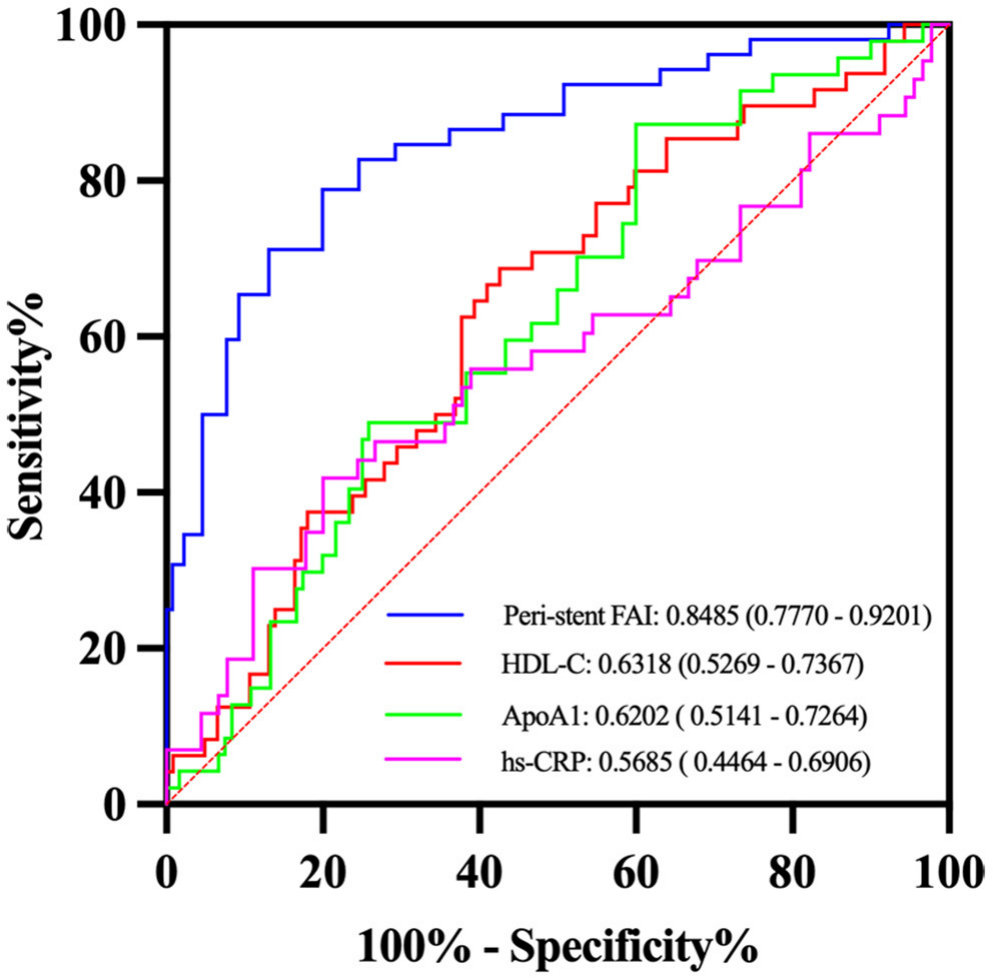
Receiver operator characteristic (ROC) curves analysis of selected variables for detection of ISR. The peri-stent FAI had the best performance with an AUC value of 0.85.

The ROC analysis showed that the peri-stent FAI had the highest prediction performance (AUC: 0.8485, 95% CI, 0.777–0.920) for ISR, followed by HDL-C (AUC: 0.6318 (95% CI, 0.527–0.737), ApoA1, [AUC: 0.6202 (95% CI, 0.514–0.726)] and hs-CRP for predictive performance (AUC: 0.5685, 95% CI, 0.446–0.691) (Figure 5). The cutoff value of peri-stent FAI was 82.5 HU (82.69% sensitivity and 73.85% specificity).

### Subgroup Analysis of the Three Major Coronary Arteries

**Figure 6:**
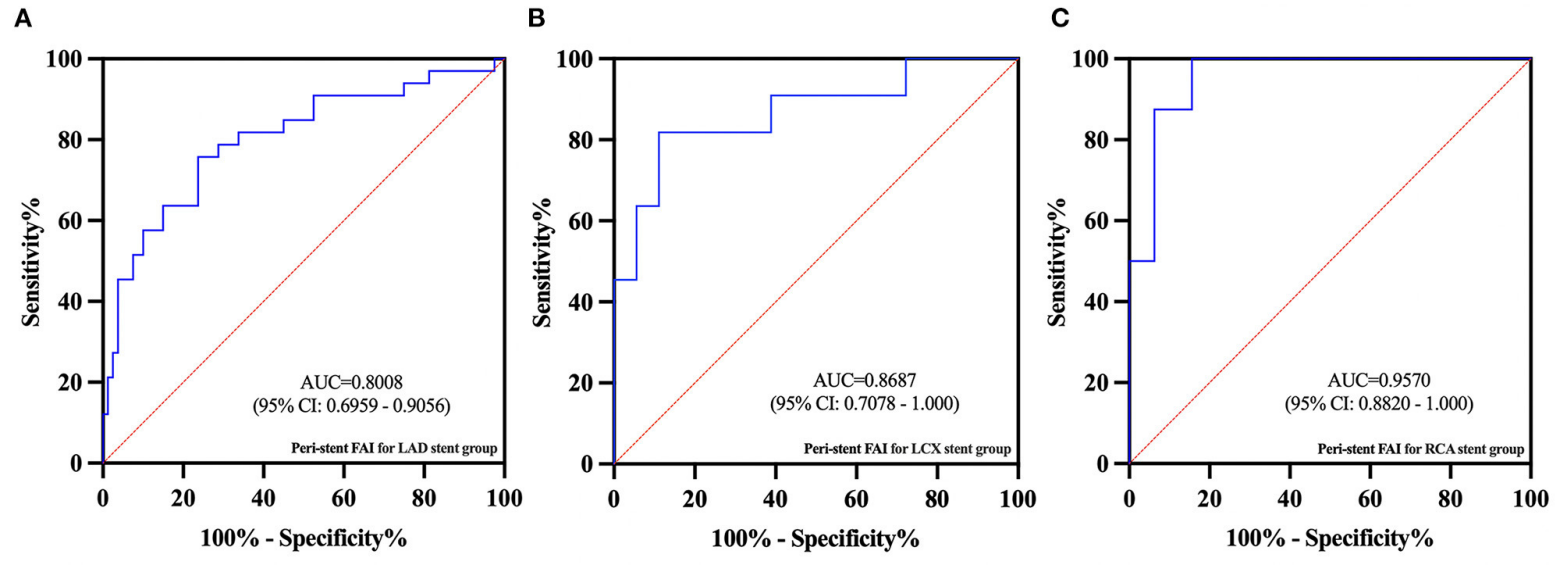
In the subgroup ROC analysis, the AUC values for ISR predicted by peri-stent FAI in the LAD **(A)**, LCx **(B)**, and RCA **(C)** subgroups were 0.80, 0.87, and 0.96, respectively.

In subgroup analysis, there were 73 patients in the LAD subgroup (40 non-ISR and 33 ISR), 20 patients in the LCx subgroup (9 non-ISR and 11 ISR), and 24 patients in the RCA subgroup (16 non-ISR and 8 ISR). In the LAD subgroup, the peri-stent FAI was significantly lower for the non-ISR group (−86.1 ± 6.0 HU) when compared with the ISR group (−79.5 ± 5.8 HU) (*p* < 0.001). In the LCx and RCA subgroups, the peri-stent FAI also showed significant differences between the non-ISR and ISR participants (−85.9 ± 6.0 HU vs. −75.7 ± 7.0 HU, *p* < 0.01 and −90.9 ± 9.9 HU vs. −75.2±5.4 HU, *p* < 0.001, respectively). The complete subgroup analyses results are presented in Table 5. The AUC values for ISR prediction were 0.80, 0.87, and 0.96, for LAD, LCx, and RCA subgroups respectively (Figure 6).

**Table 5:** Comparison of the parameter in three major coronary artery subgroups.

**Subgroup**	**Non-ISR/ISR**	**Peri-stent FAI (HU)**	***P*** **-value**
LAD stent	Non-ISR (*n* = 40)	−86.1 ± 6.0	<0.001***
	ISR (*n* = 33)	−79.5 ± 5.8	
LCx stent	Non-ISR (*n* = 9)	−85.9 ± 6.0	0.003**
	ISR (*n* = 11)	−75.7 ± 7.0	
RCA stent	Non-ISR (*n* = 16)	−90.9 ± 9.9	<0.001***
	ISR (*n* = 8)	−75.2 ± 5.4	

Values are given as mean ± SD (range) or n (%), “**”, “***” indicate significance at p < 0.01 and p < 0.001 respectively.

*ISR, in-stent restenosis; FAI, fat attenuation index; LAD, left anterior descending coronary artery; LCx, left circumflex coronary artery; RCA, right coronary artery*.

### Correlation of Peri-Stent FAI Values With ISR Severity

**Figure 7:**
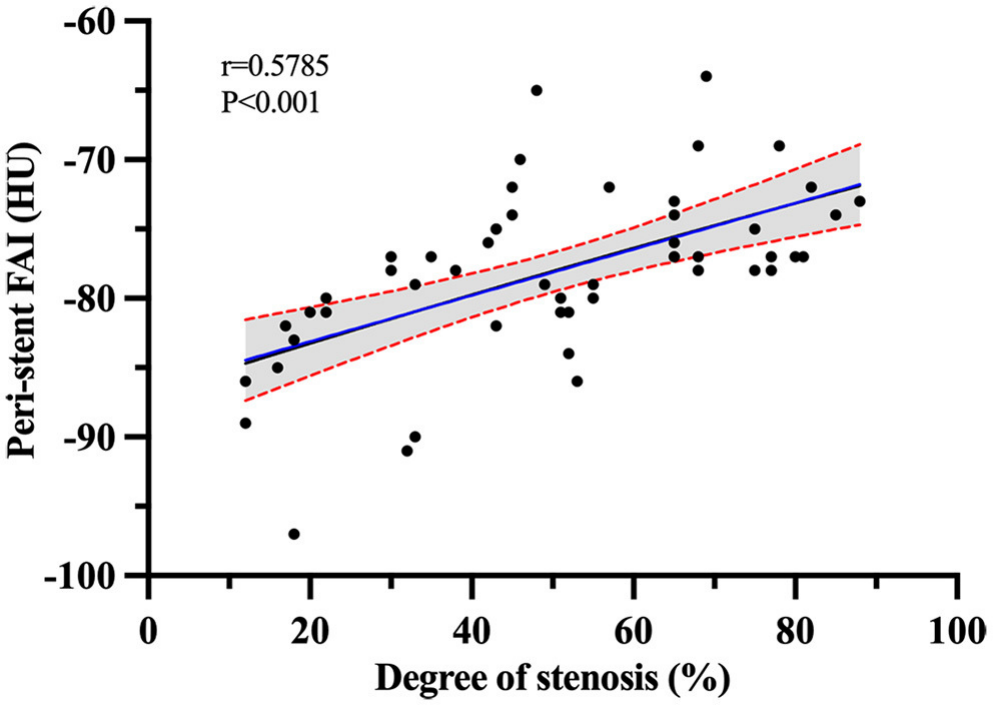
Pearson r correlation analysis between ISR severity and peri-stent FAI values in the ISR group.

Peri-stent FAI had the term moderately positive correlation with ISR severity with a Pearson correlation coefficient of *r* = 0.579, *P* < 0.001 (95% CI, 0.36–0.74) (Figure 7).

## Discussion

To the best of our knowledge, this is the first study evaluating the value of peri-stent FAI to detect PVAT inflammation around coronary artery stents and its correlation with ISR. Our findings indicate that as a CCTA-derived biomarker of coronary inflammation based on quantification of PVAT attenuation shifts (20), peri-stent FAI can be used as an independent predictor for ISR after stent implantation. Notably, this significant difference goes beyond hematological and biochemical parameters and has an important clinical value.

Although the development of DESs with antiproliferative drugs reduced the incidence of ISR when compared with BMSs, the incidence of ISR still ranges from 5 to 10% (21). In addition, the incidence of late stent thrombosis in DESs was significantly higher than that in BMSs due to delayed arterial healing (22, 23). Both BMS-ISR and DES-ISR have the same traditional mechanisms for pathogenesis, including biological, mechanical, and technical factors (24). Although DESs minimize the neointimal proliferation and improve the healing process after stent implantation compared to BMSs, high sensitivity to polymers and drugs, local inflammation, and delayed healing may still cause neointimal formation (25).

ISR may be caused by various mechanisms, including inflammation and oxidative stress. Inflammation is considered to play a key role in the ISR process (5, 6). Optical coherence tomography (OCT) studies had revealed that early ISR was characterized by homogeneous neointimal hyperplasia, while late ISR appeared as neoatherosclerosis with thin-cap fibroatheroma and lipid-rich neointima (26). Inflammation has long been implicated in atherogenesis and this has been confirmed by recent clinical trials; therefore, inflammation is also known as an important factor in the restenosis process (27–29). Histological studies from the retrieved ISR tissues revealed the substantial inflammation full of foamy macrophages and T-cell infiltration (30). Pathology studies also showed that although second-generation DESs suggest less inflammation but similar rate of neoatherosclerosis compared to first-generation ones (31). The inflammatory response processes include platelet deposition, leukocyte recruitment, and smooth muscle cell migration and proliferation (32). PVAT exerts paracrine effects on the vascular wall, which promote atherosclerosis through outward-to-inward signaling (33).

Positron emission tomography (PET) imaging is the gold standard in evaluating PVAT inflammation which is assessed by measuring the uptake of ^18^fluorodeoxyglucose. Still, its use is limited by its high exposure, high cost, and low clinical availability (34). CCTA is a more widely available modality that can detect PVAT changes as a response to inflammation and provide accurate information on ISR. Therefore, it is an ideal non-invasive tool that could be used to follow-up patients following the implantation of coronary artery stents (9, 35). The intravascular inflammatory response alters the composition of PVAT, shifting its FAI value from the lipid (closer to −190 HU) to the aqueous phase (closer to −30 HU), which had been demonstrated by histology (9). Previous clinical studies showed a significant association between CAD and higher perivascular FAI values. FAI changes were identified as a key factor contributing to the development of acute coronary syndromes. The increase of perivascular FAI values was associated with the development of coronary atherosclerotic plaques and acute myocardial infarction (14). Our results suggest that peri-stent FAI can provide an accessible non-invasive imaging method to identify coronary inflammation. The peri-stent PVAT of patients with ISR had a higher inflammatory status level than patients with non-ISR. It should be noted that the peri-stent FAI measurement method used in our study was not adjusted for technical, anatomical, and biological factors, which may limit the clinical value. Weighted FAI measurement (FAI-score) was developed by Oikonomou et al. to correct these factors (36), providing a more standardized metric of coronary inflammation than FAI and would have added further value to the study.

Various easily available inflammatory and oxidative biomarkers have been used to predict ISR. The hs-CRP was identified as a systemic inflammatory marker and has been significantly associated with adverse cardiovascular outcomes (37). Seo et al. determined that increased hs-CRP expressions tended to have higher ISR rates in older patients (29). Kuwano et al. demonstrated that the baseline serum bilirubin could be an independent predictor of ISR (38). Geng et al. found that patients with higher baseline RDW were more likely to have ISR at follow-up (39). Inconsistent with these findings, in our study, only HDL-C and ApoA1 were negatively correlated with ISR risk, and hs-CRP was not correlated with ISR. The differences in hematological biomarkers between the ISR and control groups were far less evident than the differences in the peri-stent FAI. Notably, some cytokines and adipokines [e.g., tumor necrosis factor-alpha (TNF-alpha), adiponectin, leptin, interleukin-6] locally secreted by PVAT may affect vascular physiology and pathology (40). It may be more valuable to investigate these cytokines or adipokines than the liver-derived systemic inflammation markers. Elnabawi et al. found that biologic therapy such as anti-TNF-alpha, anti-interleukin for moderate to severe psoriasis was associated with reduced coronary inflammation assessed by FAI (41).

Although the sample size in this study was small in the subgroup analysis, the RCA subgroup had the highest risk for the development of ISR. This finding can be ascribed to the fact that the RCA is surrounded by more adipose tissue when compared with the other epicardial arteries. Thus, the differences in FAI values in the RCA subgroup were more evident between the ISR and the non-ISR groups (9). It is important to note that despite blooming artifacts and image noise caused by the stents in some cases, it was relatively easy to visualize the stents and measure the FAI in most cases, low variability of intra-observer in the study also proves the feasibility of measuring FAI around the stent.

Our findings also show a term moderately positive correlation between the PVAT inflammation with the severity of ISR, indicating that the peri-stent FAI had a moderate correlation with the severity of ISR. McDonald et al. have shown that a reduction in the inflammatory response may improve long-term clinical outcomes (42). Therefore peri-stent FAI could be used to monitor the progression of ISR and the effectiveness of anti-inflammatory treatments, which would facilitate a more personalized approach to individual care.

This study has some limitations that have to be acknowledged. First, the study was based on a small sample size from a single center, limiting the generalizability of the research findings. Extensive prospective studies are needed to confirm the impact of peri-stent FAI changes on the ISR severity and progression. Furthermore, studies have shown that the clinical presentation, pathogenesis, lesion morphology, and response to intervention between the BMS-ISR and DES-ISR differ significantly (43). In addition, other stent characteristics may have an impact on the predictive value of FAI changes. However, due to the retrospective limited sample size, it was not possible to evaluate the differences between different types of stents and different polymer-coating types of DESs, and no more comprehensive data about cytokines and adipokines were available. Furthermore, the impact of different ISR components on the degree of PVAT inflammation and weighted FAI measurement were not evaluated in this study, highlighting the need for further studies. Finally, the judgment on ISR in this study was based on CCTA. Although CCTA is deemed an appropriate non-invasive angiographic tool for the follow-up of patients with ISR (35), digital subtraction angiography (DSA) is still the gold standard. Future studies should therefore compare the accuracy of our method with DSA.

## Conclusion

Our retrospective study has shown that the ISR is associated with the inflammation of peri-stent PVAT. Peri-stent FAI can facilitate ISR identification, and its use is therefore recommended as an independent non-invasive biomarker to predict ISR occurrence after PCI with stenting placement.

## Data Availability Statement

The original contributions presented in the study are included in the article/supplementary material, further inquiries can be directed to the corresponding author.

## Ethics Statement

The studies involving human participants were reviewed and approved by Medical Ethics Committee of Affiliated Hospital of Nanjing University of Chinese Medicine. Written informed consent for participation was not required for this study in accordance with the national legislation and the institutional requirements. Written informed consent was not obtained from the individual(s) for the publication of any potentially identifiable images or data included in this article.

## Author Contributions

BQ and ZW designed the study, wrote the manuscript, and conducted statistical analysis. ZL conducted data inspection and validation. HZ and YL revised the manuscript. HW conducted data collection. All authors contributed to the article and approved the submitted version.

## Funding

The present study has received funding from the Postgraduate Research & Practice Innovation Program of Jiangsu Province (grant number: SJCX21_0784).

## Conflict of Interest

The authors declare that the research was conducted in the absence of any commercial or financial relationships that could be construed as a potential conflict of interest.

## Publisher's Note

All claims expressed in this article are solely those of the authors and do not necessarily represent those of their affiliated organizations, or those of the publisher, the editors and the reviewers. Any product that may be evaluated in this article, or claim that may be made by its manufacturer, is not guaranteed or endorsed by the publisher.
